# Establishment and molecular characterization of a human ovarian clear cell carcinoma cell line (FDOV1)

**DOI:** 10.1186/s13048-018-0429-5

**Published:** 2018-07-09

**Authors:** Wei Jiang, Shuang Ye, Libing Xiang, Wentao Yang, Tiancong He, Xuan Pei, Lin Guo, Huijuan Yang

**Affiliations:** 1 Department of Gynecologic Oncology, Fudan University Shanghai Cancer Center, Shanghai, 200032 China; 2 Department of Pathology, Fudan University Shanghai Cancer Center, Shanghai, 200032 China; 3 Department of Oncology, Shanghai Medical College, Fudan University, Shanghai, 200032 China; 4 Department of Clinical Laboratory, Fudan University Shanghai Cancer Center, Shanghai, 200032 China

**Keywords:** Ovarian clear cell carcinoma, Cell line, FDOV1, ARID1A, PIK3CA, SPOP, ZNF217 amplification

## Abstract

**Background:**

Ovarian clear cell carcinoma is a distinct histologic subtype with grave survival. The underlying molecular mechanism is not fully elucidated. However, we don’t have many cell lines, which are useful experimental tools for research. We describe the establishment and characterization of a new ovarian clear cell carcinoma cell line from a Chinese patient.

**Results:**

FDOV1 has been subcultured for more than 80 generations. Monolayer cultured cells are polygonal in shape, showing a transparent cytoplasm full of vacuoles. The number of chromosomes ranges from 45 to 90. FDOV1 cells produces CA-125, but not CA-199. The cells could be transplanted and produced tumors mimicking the donor tumor morphologically and immunohistochemically. Whole exome sequence showed both FDOV1 and tissue block harbored PIK3CA H1047R mutation and ARID1A frameshift mutations (p.L2106 fs, p.N201 fs). More interestingly, we observed SPOP mutation (p.D82H) and ZNF217 (chromosome 20q13) amplification in FDOV1, which are quite novel.

**Conclusions:**

Only a few patient-derived ovarian clear cell carcinoma cell lines have been reported in the literature. FDOV1 is the very first one, to the best of our knowledge, from a Mainland Chinese patient. It showed infinite multiplication until now and tumorigenicity in vivo. FDOV1 has co-existing PIK3CA and ARID1A mutations. It also harbored SPOP mutation and ZNF217 amplification, which would probably be a good model for exploring the molecular mechanism of ovarian clear cell carcinoma.

**Electronic supplementary material:**

The online version of this article (10.1186/s13048-018-0429-5) contains supplementary material, which is available to authorized users.

## Background

Epithelial ovarian carcinoma is the most lethal gynecologic malignancy. Ovarian clear cell carcinoma (OCCC) is the second most common histologic subtype, accounting for 5–25% of all ovarian cancer depending on geographic location [1, 2]. It is well acknowledged that OCCC is more commonly seen in Asia women [3, 4]. OCCC represents a great challenge due to its disease aggressiveness and chemotherapy resistance. The grave survival and lack of effective treatment prompt us to investigate the underlying molecular mechanism of OCCC.

The two most widely reported gene mutations in OCCC are AT*-*Rich Interactive Domain-containing protein 1A (ARID1A) and Phosphoinositide-3-kinase Catalytic Alpha (PIK3CA), representing around 50% [5, 6] and 40% [7, 8] of all cases, respectively. Further studies supported that loss of ARID1A expression frequently coexisted with PIK3CA mutations [9]. What’s more, coexistent ARID1A-PIK3CA mutations might promote ovarian clear cell tumorigenesis through synergic effects [10, 11]. The breakthrough finding of ARID1A mutation has renewed the interest in elucidating the molecular pathways of OCCC [2], which still remains less well-understood than that of high-grade serous carcinoma.

Cancer cell lines are affordable models for genetic and molecular profiles, reflecting the characteristics of the origin tumor. According to documentations till now, 16 OCCC cell lines were established and their characteristics have been reported [12–25], added ES-2 and TOV-21G, two commercial available cell lines, which characteristics and genetic features were obtained from ATCC database (http://www.atcc.org) and COSMIC database (https://cancer.sanger.ac.uk/cell_lines), respectively. The majority of the established cell lines were derived from Japanese patients. Besides, only one OCCC cell line TOV-21G was found with PIK3CA and ARID1A mutation. We describe here the establishment and characterization of a novel cell line (FDOV1) from a Chinese patient that harbors coexistent ARID1A-PIK3CA mutations.

## Results

### Morphology and growth characteristics

**Fig. 1:**
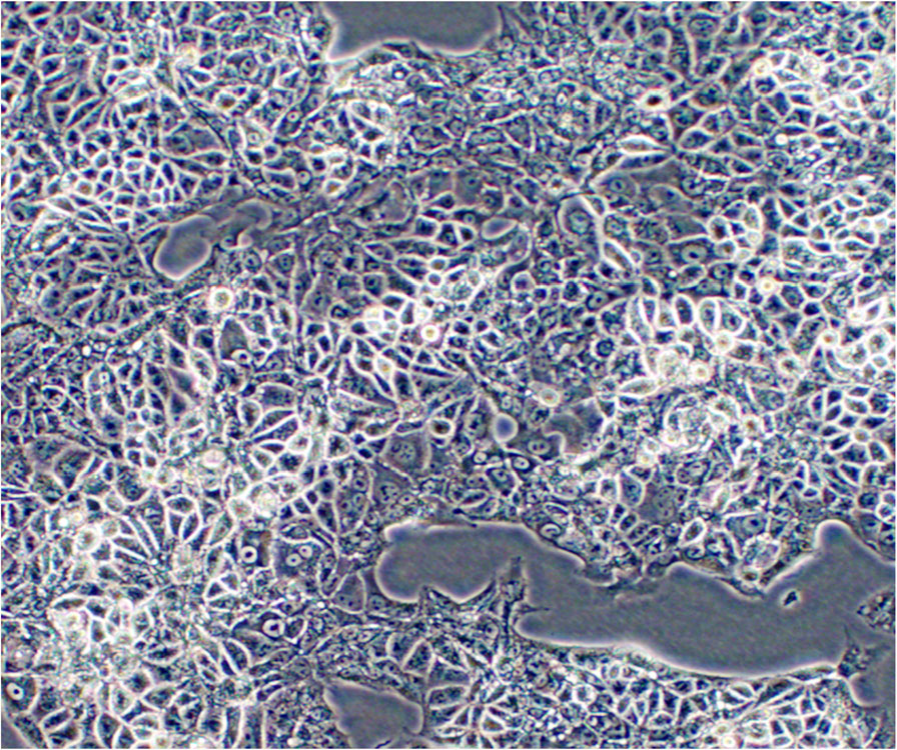
Phase contrast microscopy (magnification × 100)

Up to now, more than 80 serial passages have been carried out successively. FDOV1 cells grew in the form of an adherent monolayer without contact inhibition (Fig. 1). Several types of cells were noted: small round cells, oval cells, polygonal spindle cells and irregular cells. The cytoplasm was characteristic of transparency and vacuolation.

**Fig. 2:**
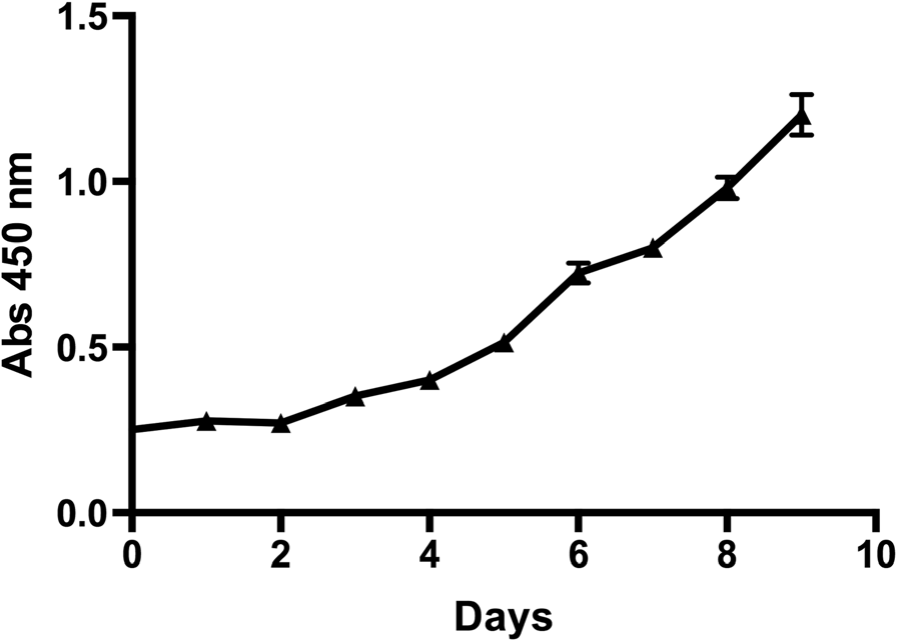
Growth curve of FDOV1 (15th generation)

**Fig. 3:**
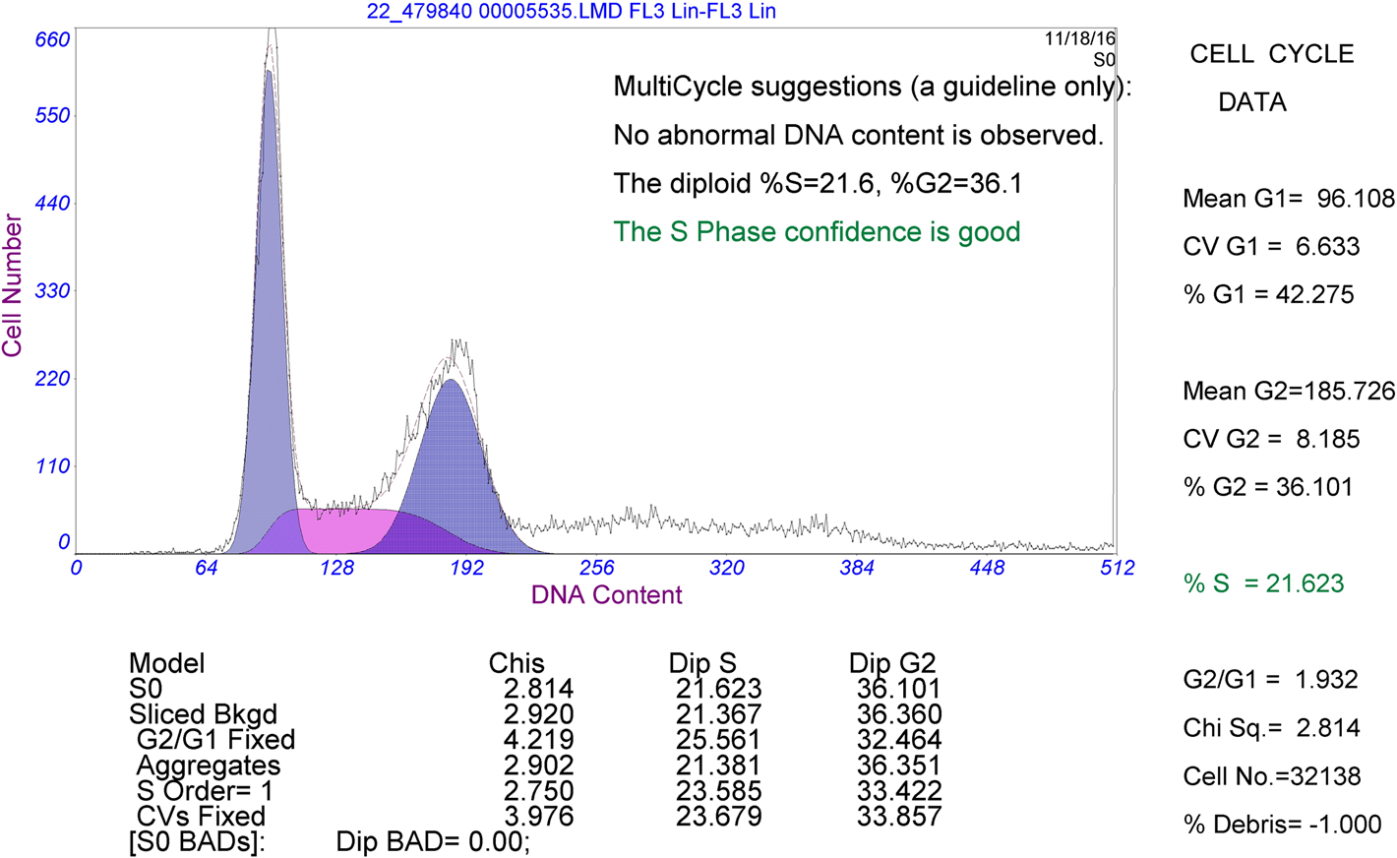
Cell cycle of FDOV1 by flow-cytometry analysis

The cell growth curve was shown in Fig. 2. The population doubling time was 37.4 h, which was consistent with the low proliferation rate of OCCC. On flow cytometry analysis (Fig. 3), the cell cycle was distributed as: G1 phase, 42.3%; G2 phase, 36.1%; S phase, 21.6%.

### Chromosomal analysis

**Fig. 4:**
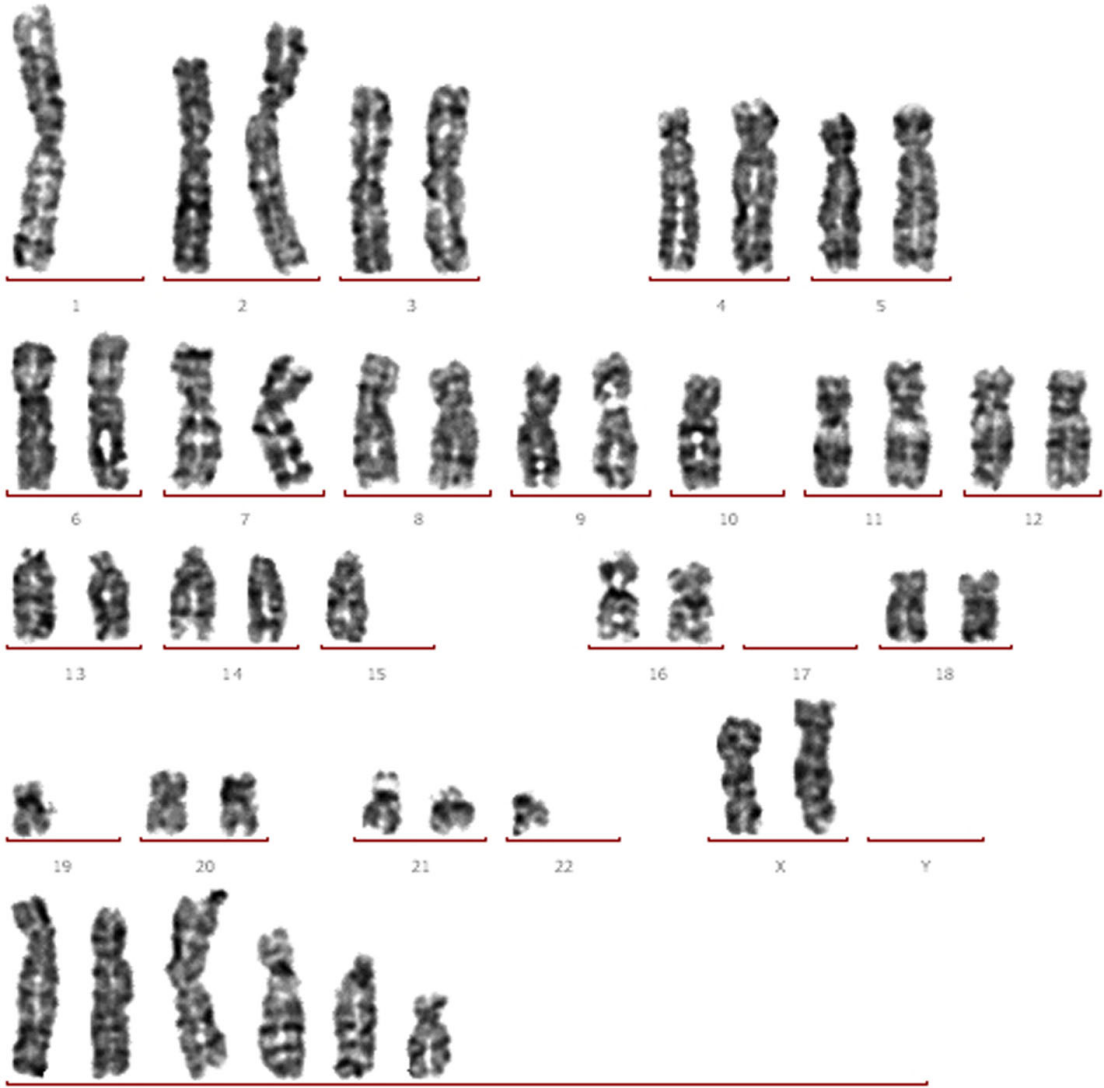
Karyotype of FDOV1 (18th generation). The additional chromosomes listed in the lowest group have severe structural abnormality, including complex translocation and deletion. It is so difficult to identify the chromosomes that we present them in the lowest group

A total of 45 metaphase cells from FDOV1 were examined for chromosome analysis. Chromosome numbers ranged from 45 to 90 (Fig. 4). The following karyotypes were observed: 2*n* = 45/46 (35, 78%); 2*n* = 47 (3, 7%); 4*n* = 86–90 (6, 13%). Chromosomal aberrations including complex translocations and deletions were noted, which were consistent with that of malignant tumors.

### Tumor markers

The results for tumor marker measurement were listed as follow: CA125, 33.7 U/ml; CA199, 0.78 U/ml; CA153, < 1.00 U/ml; CA724, 2.96 U/ml; AFP, < 0.61 ng/ml; CEA, 0.21 ng/ml; HE4, < 15 pmol/L.

### Heterotransplantation

**Fig. 5:**
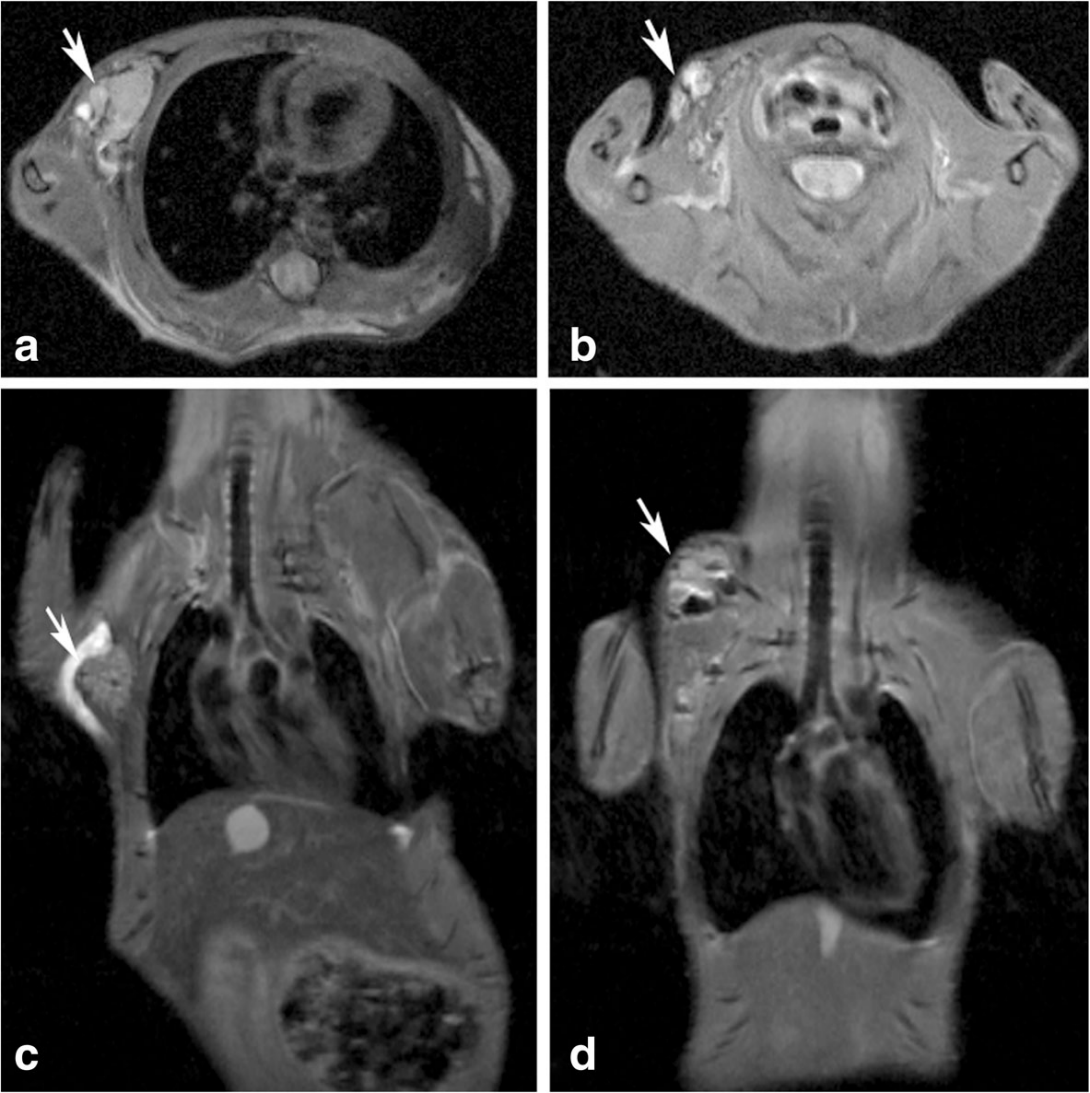
Magnetic resonance imaging of two transplanted tumors. **a** and **c** showed the horizontal plane and coronal plane of one transplanted tumor, respectively, **b** and **d** showed the horizontal plane and coronal plane of the other transplanted tumor. The tumors in the dorsal flank were pointed out by arrows

To determine tumorigenicity, FDOV1 cells were injected into the dorsal flanks of two mice. Both mice developed visible tumors 20 days after injection. Fig. 5 presents the magnetic resonance imaging (MRI) of transplanted tumors of two mice 40 days after transplantation.

### Histopathology and immunohistochemical staining

**Fig. 6:**
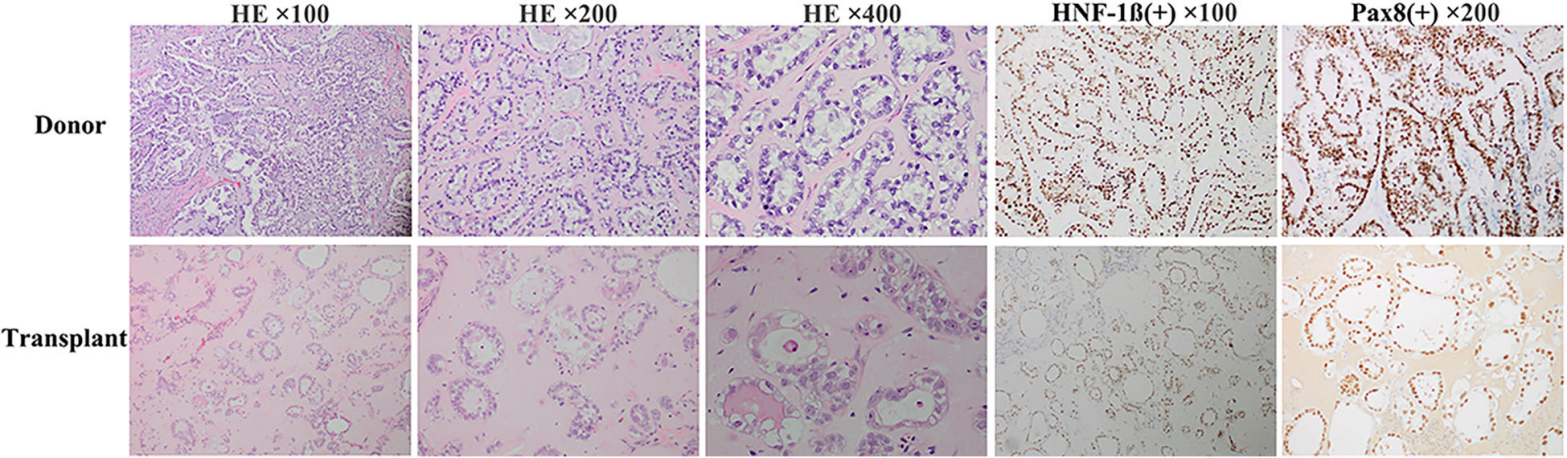
Histology and immunohistochemistry of FDOV1 cell, donor tumor and transplanted tumor

As clearly seen from Fig. 6, the xenografts morphologically mimicked the primary tumor in HE staining. Both the transplant and donor tumors showed diffuse intensive positivity for HNF-1β and Pax-8. Besides, negative Napsin A, ER and PR were observed for both tumors (results not presented).

### WES and sanger sequencing results

**Fig. 7:**
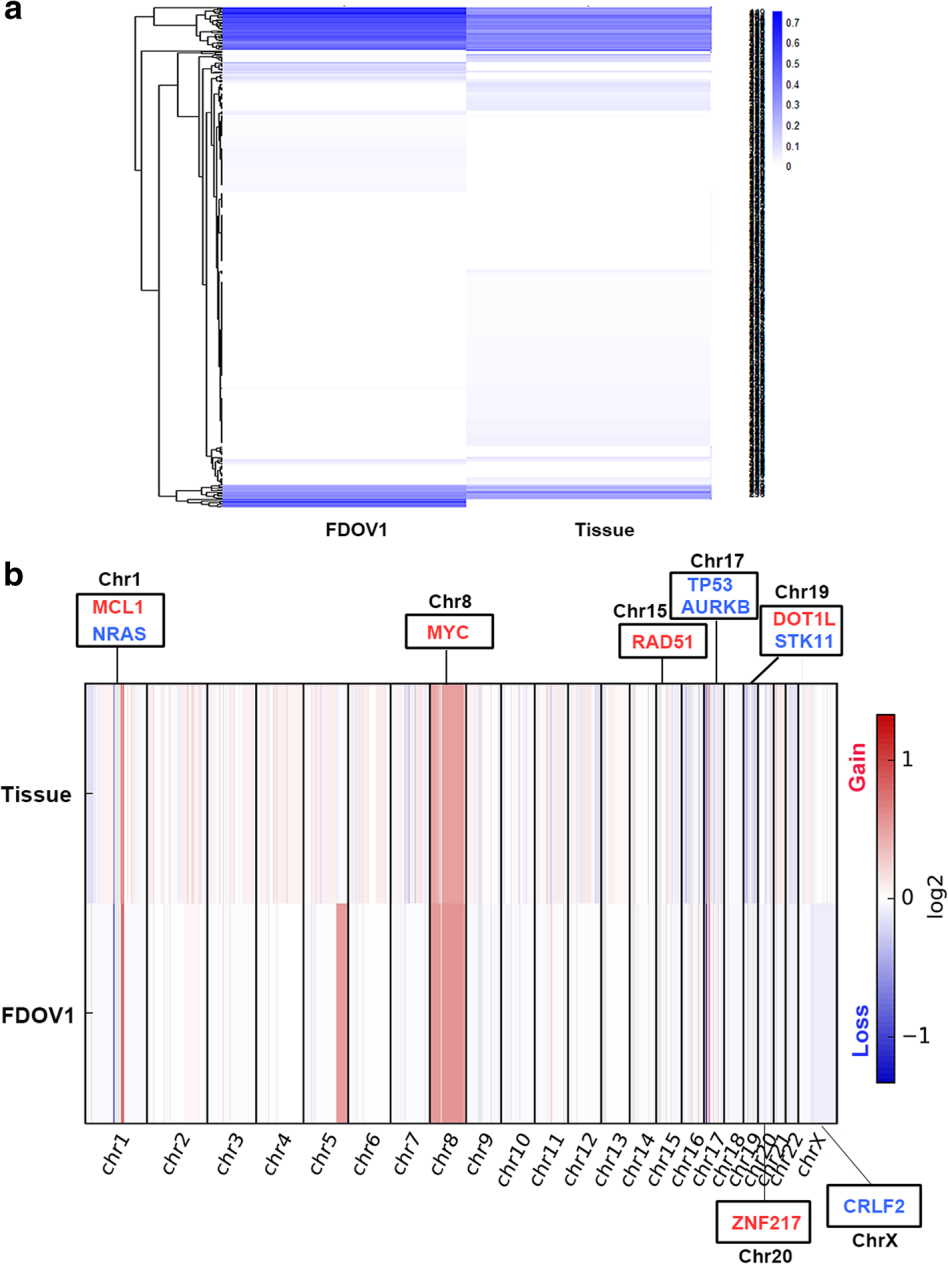
The results of whole-exome sequencing of FDOV1 and patient’s tumor tissue block. **a**. Somatic variation of FDOV1 and tumor tissue. **b**. Copy number analysis in FDOV1 and tissue. The genes in red mean copy number gain, the genes in blue mean copy number loss

**Fig. 8:**
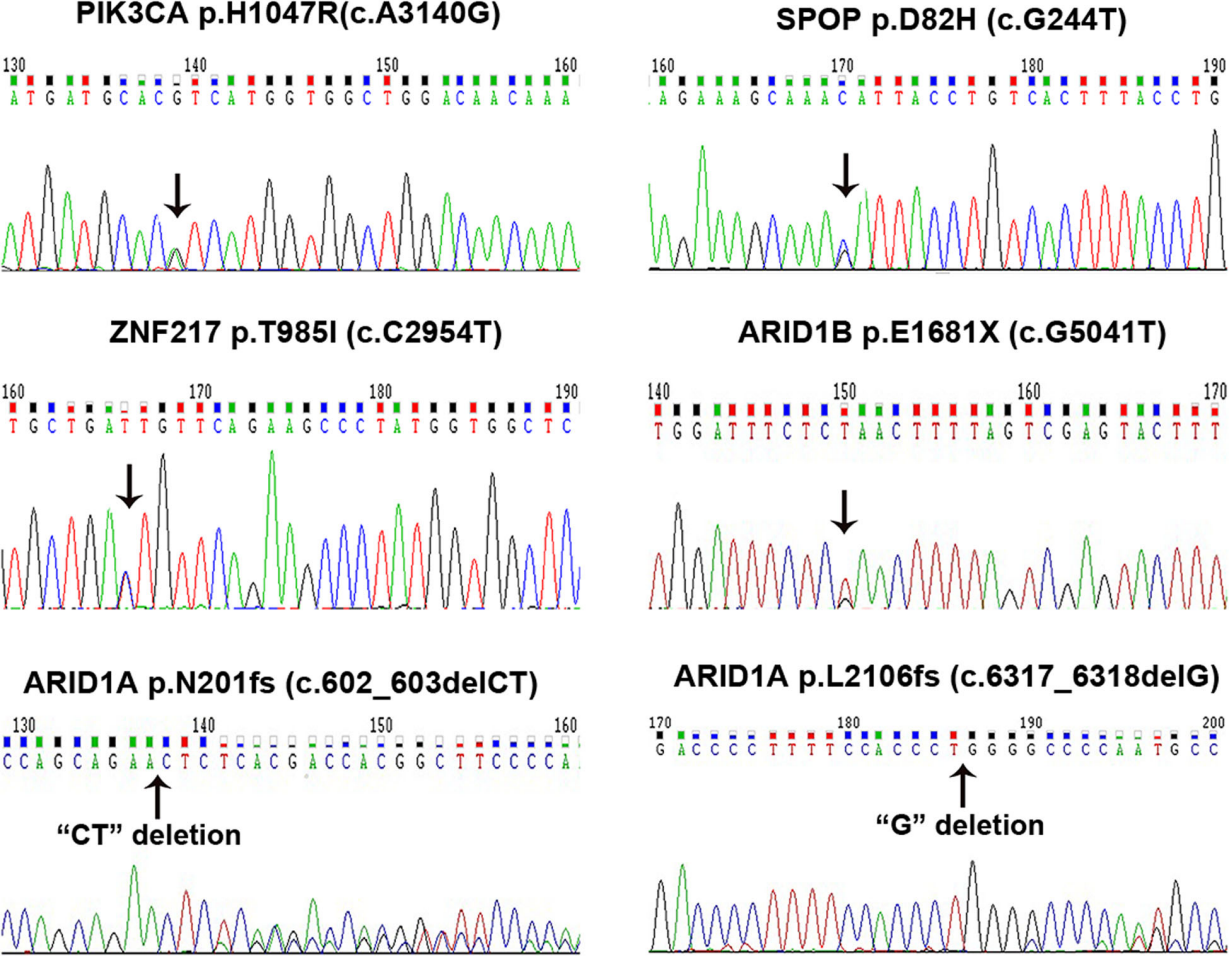
Sanger sequencing of single nucleotide variant (SNV) in FDOV1

We performed WES on FDOV1 cell, patient’s tissue block and peripheral blood. It was reported that the tumor purity of FDOV1 cells achieved 100% approximately, and that of the tumor block was 73%, which is fit for investigating the molecular aberrations, including single nucleotide variant (SNV), insertion and deletion (indel) and copy number variation (CNV). From the SNV related to cancer, it is shown that the tissue block and FDOV1 cell line shared some same somatic variations although diverse allele frequency (Fig. 7a). Interestingly, both tissue block and FDOV1 harbored PIK3CA H1047R mutation and ARID1A frameshift mutations (p.L2106 fs, p.N201 fs), which are viewed as the most important gene variations in OCCC. In addition, Both of them were found to carry SPOP (p.D82H, c.G244 T), ZNF217 (p.T985I, c.C2954T) and ARID1B (p.E1681X, c.G5041 T) mutations (Table 1), which were further validated by Sanger Sequencing (Fig. 8). Besides, Fig. 7b showed the CNV of tissue block and FDOV1. We observed that somatic copy number was increased in ZNF217(chr.20) and MYC(chr.8), which were reported as frequent amplifications in OCCC [26, 27], as well as MCL1(chr.1), RAD51(chr.15), DOT1L(chr.19), while copy number was decreased in TP53(chr.17), which was documented as a common copy number variant in OCCC [26], as well as NRAS(chr.1), AURKB(chr.17), STK11(chr.19) and CRLF2(chr.X) (Table 2).

**Table 1:** Single nucleotide variant (SNV) of FDOV1 by whole-exome sequencing(WES)

Gene	Pdot	Tissue	FDOV1
PIK3CA	p.H1047R	0.372	0.369
SPOP	p.D82H	0.36	0.5
ZNF217	p.T985I	0.329	0.46
ARID1A	p.L2106 fs	0.285	0.311
ARID1B	p.E1681X	0.277	0.588
ARID1A	p.N201 fs	0.271	0.339
FOXP1	p.S650delinslS	0.081	
NOTCH2	p.P6fs	0.081	
KMT2C	p.K2797 fs	0.048	
MAP3K1	p.941_942del	0.039	
TGFBR2	p.E125fs	0.034	
KMT2C	p.T820I	0.033	
ABL1	p.605_605del	0.029	
ROCK2	p.R1295G	0.026	
KMT2D	c.8366 + 1G > T	0.02	
ERBB4	p.G565 V	0.017	
KMT2C	p.D319Y	0.014	
CTNNA1	p.M721 fs	0.013	
KMT2C	p.R4418S		0.028
KMT2D	p.3860_3861del		0.02
RNF43	p.R145L		0.018
KMT2A	p.A1253S		0.012

**Table 2:** Copy number variant (CNV) of FDOV1 by whole-exome sequencing (WES)

	Chr	Tissue	FDOV1
Upregulated Genes			
ZNF217	chr20	0.75	0.91
MYC	chr8	0.50	0.89
DOT1L	chr19	0.78	1.12
MCL1	chr1	0.48	0.77
RAD51	chr15	0.47	0.94
Downregulated Genes			
NRAS	chr1	−0.65	−1.01
TP53	chr17	−0.55	− 1.04
AURKB	chr17	−0.55	−1.04
STK11	chr19	−0.64	−1.05
CRLF2	chrX	−1.38	−1.41

The number in column of tissue and FDOV1 represent the log2 (sample/germline), consequently, the number more than 0 suggests amplified in copy number, less than 0 suggests decreased in copy number

## Discussion

Ovarian clear cell carcinoma, as a subtype with distinct clinical behavior, is notorious for its poor survival and resistance to conventional platinum-based chemotherapy. An international phase III clinical trial was conducted to investigate the addition of irinotecan as adjuvant chemotherapy for patients with advanced-stage [28]. Unfortunately, no significant survival benefit was found. Given the limited benefit from cytotoxic drugs, there is now great interest in the development of molecular targeted therapy, which requires investigation of a number of areas in the laboratory [3]. Better understanding of the underlying molecular mechanism is of importance.

According to documentations, there were 16 ovarian clear cell carcinoma cell lines reported so far, in addition, TOV-21G, ES-2, which are commercially available from American Tissue Culture Center, and FDOV1. Their characteristics were compared in Table 3. Firstly, most of the OCCC cell lines were from Japan, while FDOV1 is the only one derived from a Chinese mainland woman. It has been demonstrated that the same kind of carcinoma from different race even country tends to have distinct characteristics, especially genetic characteristics [29–31]. We postulated that genetic background might be partly the reason for the marked geographic difference in OCCC. Therefore, the establishment and characterization of a novel cell line derived from Chinese patients is of significance. Secondly, all cell lines were derived from ovary (10/19), ascites (6/19), and metastatic or recurrent site (3/19), which implied good materials for primarily culture. Lastly, as previously mentioned, ARID1A and PIK3CA mutations are the two most commonly reported. Interestingly, it is found that ARID1A–PIK3CA mutational co-occurrence (33%) is very high in ovarian CCC [11]. Research work by Chandler et al. supported that a genetic epistasis model wherein ARID1A and PIK3CA mutations cooperate and ovarian cancer can arise only when these genes are co-mutated in the mouse ovarian surface epithelium [11]. Coexistent ARID1A–PIK3CA mutations might be a major driver of OCCC in humans. Among 19 OCCC cell lines, only FDOV1 and TOV-21G carried coexistent ARID1A–PIK3CA mutations. The character of concurrent ARID1A–PIK3CA mutations of FDOV1 might help to better understand the underlying mechanism and even to explore the activity of dual inhibitors in vitro. However, the cell lines established before 2000 lack genetic information due to testing techniques. Besides, some new molecular features found by WES in FDOV1 requires more attentions.

**Table 3:** Cell lines of ovary clear cell carcinoma

	Cell line	Country	Age	Material	DT	Chromosome number	Transplantability	Gene characteristics
1	HUOCA-II (1987)	Japan	51	Ovary	24,28	46	Yes	
2	RMG-I (1988)	Japan	34	Ascites	60	47	Yes	
3	OCC1 (1990)	Hong Kong	47	Ascites	36,38	70–77	Yes	
4	RMG-II (1991)	Japan	53	Ascites	58	hypertetraploid	No	
5	TOV-21G(1991)^a^	French-Canadian descent	62	Ovary	36	47	Yes	ARID1A p.Q758fs^a^75, ARID1A p.Y551fs^a^72, KRAS p.G13C, PIK3CA p.H1047Y
6	ES-2(1991)^a^	Black	47	Ovary	24	66- 88	Yes	BRAF p.V600E, MAP2K1 p.D67N
7	OVISE (1995)	Japan	40	Metastatic tumor	60–70	59–65	Yes	
8	OVTOKO (1995)	Japan	78	Metastatic tumor	60–70	76–83	Yes	
9	JHOC-5 (1999)	Japan	47	Ovary	52	74–85	No	
10	JHOC-6 (1999)	Japan	43	Recurrent tumor	70	46–49	Yes	
11	SMOV-2 (1999)	Japan	46	Ovary	48.2	85–92	Yes	P53 mutation (−)
12	TAYA (2002)	Japan	43	Ascites	50	69–74	No	P53 mutation (codon 132 in exon 5), PTEN mutation(−)
13	RMG-V (2005)	Japan	52	Ascites	15.5	77–85	No	
14	TU-OC-1 (2013)	Japan	65	Ovary	38.4	64–90	Yes	PIK3CA E542K mutation
15	TU-OC-2(2016)	Japan	68	Ovary	37.5	41–96	No	PIK3CA mutation (−), TP53 mutation (−) ARID1A loss
16	HCH-1 (2016)	Japan	67	Ovary	48.7, 66.4	39–44	Yes	PIK3CA mutation (−), P53 mutation(−), PTEN mutation(−), KRAS mutation(−)
17	NOCC(2016)	Japan	48	Ascites	29	60–83	Yes	
18	HCH-3 (2017)	Japan	41	Ovary	82	78–87	Yes	KRAS mutation (+), TP53 mutation(+)
19	FDOV1(2017)	China mainland	67	Ovary	37.4	45–90	Yes	PIK3CA p.H1047R, ARID1A p.L2106 fs, ARID1A p.N201 fs, SPOP p.D82H ARID1B p.E1681X

^a^The biological characteristics of TOV-21G and ES-2 were obtained from ATCC database (http://www.atcc.org), and the genetic variants of TOV-21G and ES-2 were obtained from COSMIC database (https://cancer.sanger.ac.uk/cell_lines), which provide more detailed and complete genetic features of cell lines

Notably, SPOP, a relatively new gene regulating DNA damage repair, is the most frequently mutated gene in prostate carcinoma [32, 33]. Mutations in SPOP lead to genomics instability and are identified as driver events resulting in tumorigenesis of prostate carcinoma via coordination with PI3K/mTOR and AR (Androgen Receptor) pathway in mouse [34, 35]. Studies on SPOP mutation in other solid carcinomas is reported in follicular and papillary thyroid cancers [36, 37], endometrial clear cell carcinoma [38, 39], gynecological carcinosarcoma [40], lung cancer [41], and colorectal cancer [42]. What is worthy of mentioning is that SPOP mutation in gynecological malignant tumor is not uncommon (18% in endometrial clear cell carcinoma, 14% in carcinosarcoma) [38, 40]. In OCCC, the mutation rate of SPOP is 11.1% (1/9) according to COSMIC (Catalogue Of Somatic Mutations In Cancer) database. However, few relevant functional studies are documented in OCCC. Thus, FDOV1, a novel cell line harbored SPOP mutation, is an ideal tool for functional study. In addition, it is suggested that SPOP mutation might represent sensitivity to DNA damaging agents such as PARP inhibitor in prostate carcinoma [35]. Consequently, whether it is indicated a similar effect on OCCC still needs further investigation.

Chromosome 20q13 ZNF217 (Zinc Finger Protein 217) locus amplification, which has found in FDOV1, is one of common molecuclar genetic aberrations in OCCC [43]. The frequency is reported from 20 to 36% [10, 43, 44]. It is reported that ZNF217 amplification often coexisted with loss of ARID1A expression and PIK3CA mutation [10]. Importantly, ZNF217 amplification has a correlation with shorter worse survival outcome in OCCC, and multivariate analysis suggest it is an independent prognostic factor of PFS (Progression-Free Survival) and OS (Overall survival) [43]. Still, ZNF217 amplification has an adverse prognostic implication in breast and gastric cancer [45–47]. However, the underlying mechanism is still tensely studied. FDOV1 will be an good cell line for mechanism research in vitro.

## Conclusions

An ovarian clear cell carcinoma cell line named FDOV1 was established from a Mainland Chinese patient**.** FDOV1 showed fast and unlimited multiplication until now and tumorigenicity in vivo. In addition, WES showed that it harbors concurrent ARID1A–PIK3CA mutations and SPOP mutation, also with ZNF217 amplification, which will be an ideal tool for mechanism research of ovarian clear cell carcinoma.

## Methods

### Medical history

A 67-year-old woman was admitted into Fudan University Shanghai Cancer Center with the chief complaint of palpable abdominal mass in May 2016. Ultrasound revealed a large pelvic mass (14 × 10 cm) with mixed components and a hyperechoic mural nodule (4 × 3 cm). Serum tumor markers including carbohydrate antigen (CA) 125 and CA199 were not elevated. Intraoperative findings were only significant for a cystic-and-solid mass (15 cm in diameter) in the left adnexa. Frozen pathology was highly suspicious of ovarian clear cell carcinoma. The patient underwent comprehensive staging surgery and the final stage was FIGO (International Federation of Gynecology and Obstetrics) Ia. She gave written informed consent before surgery according to institutional guideline (FUSCC 050432–4-1212B). Six cycles of TC regimen (paclitaxel + carboplatin) chemotherapy were administered. She was followed up every 3 months after treatment. The patient was admitted into outside hospital with chief complain of intestinal obstruction in May 2017. She developed multiple sites of venous thromboembolism and cerebral infarction. Unfortunately, she died 1 month later with no chance of anti-cancer treatment.

### Establishment of cell line

The fresh tissue (1 cm^3^) from primary ovarian tumor was obtained during comprehensive staging surgery, and suspended in phosphate buffer solution (PBS) to get rid of necrotic and connective tissue. The material was then finely minced into 1-mm^3^ tissue blocks and placed in 10-cm cell culture dishes (Corning, NY, USA) containing Medium 199 medium (HyClone, Thermo Scientific, USA) with 10% fetal bovine serum (Gibco, Life technologies, USA), 100 IU/ml penicillin (HyClone, Thermo Scientific, USA) and 50 μg/ml streptomycin (HyClone, Thermo Scientific, USA). The cells were then incubated in a humidified atmosphere containing 5% CO2 at 37 °C. After repeated passage, a new cell line of FDOV1 was successfully established with more than 80 generations. FDOV1 cells have been sent to China General Microbiological Culture Collection Center for preservation (No. 13812) in March 2017.

### Growth characteristics

Cell proliferation and growth curve was determined by Cell Counting Kit-8 (CCK-8) test. FDOV1 cells (2 × 10^3^ cells/100 μl) were seeded in 96-well plates in sextuplicate. Ten days later, 100 μl mixture (CCK-8/medium 1:9) were added into the wells for incubation by 2 hours. We then measured the Optical Density (OD) at 450 nm (OD_450_) by a microplate reader (Synergy H4, Bio-Tek).

### Cell cycle analysis by flow cytometry

Resuspended FDOV1 cells (1 × 10^6^ cell) were kept overnight in 3 ml 75% ethanol at − 20 °C. After treatment, the cells were collected and stained with propidium iodide solution for 20 min at 4 °C in darkness. We then assessed the cell cycle by flow cytometer (BD Biosciences, San Jose, CA, USA).

### Chromosome analysis

The cells in exponential phase were treated with 0.25 μg/ml colchicine for 6 hours and kept overnight at 37 °C. We then treated the cells with 0.1% trypsin solution for 15 s at room temperature and stained them with 3% Giemsa for G-band karyotyping. After examing 45 metaphases, the histograms of the chromosomal distribution were determined.

### Tumor markers

A total of 2 × 10^6^ cells were cultured for 4 days in total. The medium was changed and collected every 2 days, then were sent for tumor marker detection by chemiluminescence immunoassay. Detected markers included CA 125, CA 199, CA 153, CA 724, AFP, CEA and HE4.

### Heterotransplantation

All the procedures were approved by the Department of Laboratory Animal Science in Fudan University. For in vivo studies, 4–6 weeks old NOD/SCID mice (Shanghai SLAC Laboratory Animal, Shanghai, China) were maintained under sterile conditions. FDOV1 cells (1 × 10^7^ cells, passage 18) were injected subcutaneously into the dorsal flanks of two mice. Visible tumors were noted in both mice 20 days after transplantation.

### Histological analysis and immunohistochemistry

Transplant tumors were fixed in 10% buffered formalin for 24 h and embedded in paraffin. Hematoxylin and eosin (HE) were used to stain 4-μm sections. The markers used for immunohistochemistry were listed as follows: HNF-1β (Sigma, CA, USA), Napsin A (Abcam, Cambridge, UK), Pax-8 (Cellmarque, CA, USA), ER (Roche, Basel, Swiss) and PR (Roche, Basel, Swiss). Microscopic slides were reviewed by a senior gynecology-dedicated pathologist (Prof. Yang).

### Whole-exome sequencing (WES) of FDOV1 cell and patient’s tumor tissue

The WES was performed by 3D Medicines Corporation and the specific method was the same as a previous publication [48].

### Sanger sequencing

Mutations of the specific genes in FDOV1 cells were validated by Sanger Sequencing. The RNA was extracted by Trizol Methods, then reverse transcription polymerase chain reaction (RT-PCR) was performed as our previous study [49]. The primers were showed in Additional file 1: Table S1. PCR amplification was performed as follows: denaturation at 98 °C for 5 min, 30 cycle of 98 °C for 10s, 58 °C for 30s, and 72 °C for 1 min, then holding at 72 °C for 1 min, finally incubation at 4 °C. Sequencing was performed at Boshang Biotechnology Co.Ltd. (Shanghai, China).

## Additional files

Additional file 1: **Table S1.** The primer of Sanger sequence for specific genes mutation in FDOV1. (DOCX 15 kb)

## Abbreviations

AFP: Alpha fetoprotein
ARID1A: AT*-*Rich Interactive Domain-containing protein 1A
CA125: Carbohydrate antigen125
CA199: Carbohydrate antigen 199
CEA: Carcinoembryonic antigen
HE4: Human epididymis protein 4
OCCC: Ovarian clear cell carcinoma
PIK3CA: Phosphoinositide-3-kinase Catalytic Alpha
FIGO: International Federation of Gynecology and Obstetrics
TC: Paclitaxel & Carboplatin
PBS: Phosphate buffer solution
CCK8: Cell counting kit-8
OD450: Optical density (OD) at 450 nm
HE: Hematoxylin and eosin
MRI: Magnetic resonance imaging
WES: Whole-exome sequence
SNV: Single nucleotide variant
CNV: Copy number variation
indel: Insertion and deletion
ER: Estrogen receptor
AR: Androgen receptor
PR: Progesterone receptor
ZNF217: Zinc Finger Protein 217
PFS: Progression-Free Survival
OS: Overall survival
