# Supplementary material for: Establishment and molecular characterization of a human ovarian clear cell carcinoma cell line (FDOV1)
Source: J Ovarian Res. 2018 Jul 9;11:58. doi: 10.1186/s13048-018-0429-5 (PMC6038259; doi:10.1186/s13048-018-0429-5)
Supplement: Supplementary file 1 — Table S1. The primer of Sanger sequence for specific genes mutation in FDOV1. (DOCX 15 kb) [file 13048_2018_429_MOESM1_ESM.docx]

**Table S1** The primer of Sanger sequence for specific genes mutation in FDOV1

| **Gene** | **Primer** | **5'- 3'** |
| --- | --- | --- |
| PIK3CA | F | TTCGACAGCATGCCAATCTC |
|  | R | ATCGGTCTTTGCCTGCTGAG |
| SPOP | F | TGGCCCCGTAGCTGAGAGTT |
|  | R | CCTCGCAGAAGAGGGTAAGC |
| ZNF217 | F | CTGAAGTCCAGCGTGGTTGC |
|  | R | TTTGGTCGATAATGTGCATTCC |
| ARID1B | F | AAGGCGAAAGATTACCTCCA |
|  | R | CTCGTCTTCCTCGTCGTCAT |
| ARID1A(N201fs) | F | CCTACGGCTTCGGGCAAC |
|  | R | GCTGAGCGAAGGACGAAGAC |
| ARID1A(L2106fs) | F | GAACAGGACCAAGGGGTGAG |
|  | R | AGGTTGGCCAGCAGTACCAC |
